# Advances in radiation therapy dosimetry

**DOI:** 10.4103/0971-6203.54842

**Published:** 2009

**Authors:** Bhudatt Paliwal, Dinesh Tewatia

**Affiliations:** University of Wisconsin Radiation Oncology Physics 600 Highland Ave., K4/B100 - 0600 Madison, USA

**Keywords:** Dosimetry, 4DCT, motion-adaptive optimization

## Abstract

During the last decade, there has been an explosion of new radiation therapy planning and delivery tools. We went through a rapid transition from conventional three-dimensional (3D) conformal radiation therapy to intensity-modulated radiation therapy (IMRT) treatments, and additional new techniques for motion-adaptive radiation therapy are being introduced. These advances push the frontiers in our effort to provide better patient care; and with the addition of IMRT, temporal dimensions are major challenges for the radiotherapy patient dosimetry and delivery verification. Advanced techniques are less tolerant to poor implementation than are standard techniques. Mis-administrations are more difficult to detect and can possibly lead to poor outcomes for some patients. Instead of presenting a manual on quality assurance for radiation therapy, this manuscript provides an overview of dosimetry verification tools and a focused discussion on breath holding, respiratory gating and the applications of four-dimensional computed tomography in motion management. Some of the major challenges in the above areas are discussed.

## Introduction

**Figure 1:**
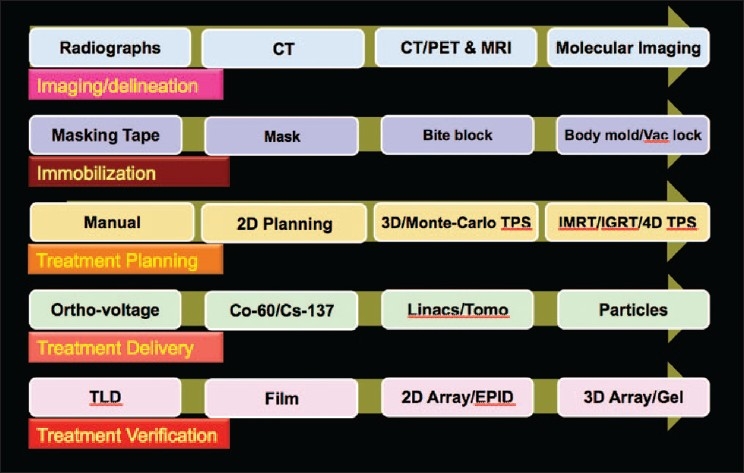
Developments in radiotherapy patient care

**Figure 2:**
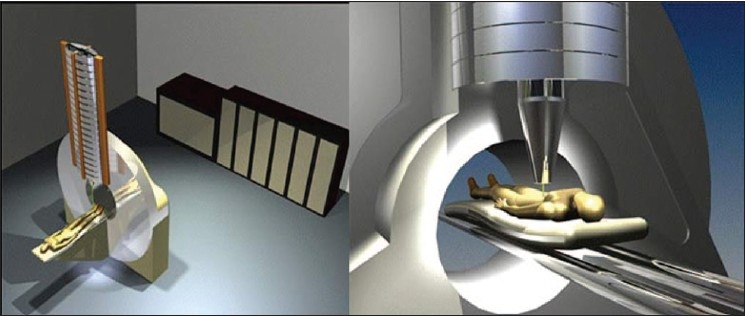
A compact proton accelerator based on Lawerence Livermore national laboratory (LLNL), Compact Particle Accelerator Corporation

**Figure 3:**
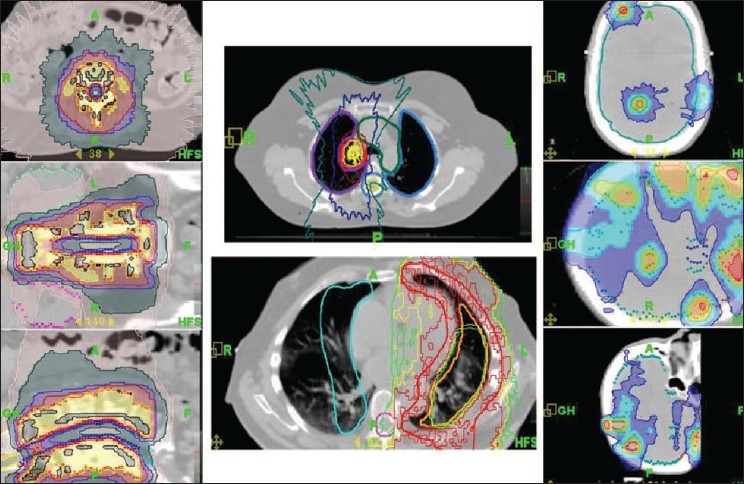
Tomotherapy-based conformal treatment plans

Medical physicists have played an important role in the technological advances in the treatment of cancers with radiation therapy. In this decade, new radiotherapy methods, such as conformal radiotherapy, chemoradiation and hyperfractionated radiotherapy, have been introduced. Technological gains [Figure 1] in radiation therapy, including IMRT and image-guided radiation therapy (IGRT), can be used to create more complex and conformal treatment plans, deliver higher target doses, use tighter margins to irradiate smaller treatment volumes and reduce toxicities to normal tissues. Along with these advancements, recently attempts are being made to implement an intensity-modulated proton therapy (IMPT) utilizing simpler and cheaper dielectric wall accelerator technology as shown in Figure 2. This technology enables protons to be accelerated to the required clinical energies - as much as 100 MeV/m - without using bending magnets or other techniques that take up space and generate unwanted radiation. The potential benefits of a correctly delivered plan include better tumor control and lower toxicities to normal tissues, the possibility to provide improved survival rate and better quality of life. A poorly delivered plan may lead to opposite outcome. These concerns become even more significant and challenging when we attempt to implement motion management–adaptive treatment planning and delivery strategies. Examples of site-specific tomotherapy-based conformal treatment plans for spine, lung and multiple brain metastases are shown in Figure 3. It is very clear from all the plans that a slight miss in dosimetric verification of such plans may defeat the whole purpose of this high conformality and higher dose for better tumor control. Pretreatment delivery and *in vivo* dosimetry performed within the target volume or close to critical structures can to some extent help address the simple but important question: have we delivered the planned treatment?

## Dosimetry verification objectives

The dosimetric goal of patient treatment has 2 components: verification of the delivered dose and verification of the patient positioning. Patient positioning verification has been significantly aided by the availability of on-board–imaging systems such as electronic portal imaging detector (EPID), megavoltage CT (MVCT) and cone beam CT (CBCT).[1,2] The verification of the dose delivered requires comparisons of measured and calculated dose distributions. Isodose lines, colored two-dimensional maps are used to visualize measured and calculated dose distributions or three-dimensional surface plots. Isodose overlays, dose difference and gamma index maps can be generated from the above data. Horizontal and vertical line profiles of these quantities can be captured interactively through specified points of the dose distribution, the dose-difference map and the map of the gamma index. Statistical analysis should be given in terms of dose difference and gamma-volume histograms. The gamma (γ) index as introduced by Low *et al.*[3] is the minimum multidimensional distance between the measurement and calculation points in a space composed of dose and physical distance coordinates, scaled by preselected tolerance limits for dose difference and distance to agreement (DTA). A value of γ ≤ 1 indicates that the two distributions agree better than the selected criteria. Moreover, γ is a continuous function rather than a simple pass-fail test. Regions where the gamma index exceeds a value of unity correspond to locations where the calculation does not meet the given criteria. Regions to be studied are those with high- and low-dose gradients. Differences between calculated and measured doses should be better than 3% and the two-dimensional gamma analysis should have a dose difference of less than 5%, 3-mm distance to agreement (DTA). Some of the related concepts used in comparative analysis are as follows:

1. Threshold: Isodose percentage line that defines the dose area to evaluate. This is a more stringent method compared to defining arbitrary boxes around regions of interest.
2. Distance to agreement: If the dose difference is not satisfied, then planned points are searched within a user-defined radius for a value that corresponds to the measured point at the center of the radius.
3. Dose difference: Planned dose is subtracted from measured dose and the difference is evaluated.

Whenever in any of the above-mentioned procedure we use film processor, it is necessary to perform its quality assurance. The gray level is dependent on the temperature of the chemicals and the concentration of developer and fixer. The processor stability and ranges of acceptable values must be tracked over time using a sensitometer. In some of the above-mentioned tests, we have to utilize a digitizer or scanner system. The digitizer response should be evaluated regularly for spatial intensity, characteristic response, and noise when there are large changes in the optical density. Data transfer should be evaluated for accuracy, wherein the pixel size and dimensions are assessed. Additionally while using the digitizer for radiochromic film analysis, we have to be careful about the light source of the digitizer.

Although the use of film for the QA is very versatile, it is also time consuming, requires additional hardware and involves a multi-step process to determine the results. It is being slowly replaced by EPID. To use an EPID for dosimetric verification, the EPID response must be characterized for dose rate, filed size, and leaf speed dependence for dynamic intensity-modulated radiotherapy (IMRT).

Recently MVCT and CBCT systems were also utilized for dose verification and implementing the dose-adaptive radiotherapy. For these systems, the establishment of the system's geometric-positioning accuracy and precision is critical for the image sets obtained from these systems to be of value in the guidance of treatment. A specific calibration procedure had to be developed to correlate the coordinate systems of the imaging and delivery systems, such that image sets from MVCT or CBCT can be used to estimate the target position with respect to isocenter. CBCT-guided shifts for all fractions of 8 prostate patients are shown in Figure 4A. A comparison of isodose distributions for a patient with IMRT plan in 28 fractions is shown in Figure 4B: (a) plan with shifts; (b) plan with maximum offsets (0.0, 0.3, -0.6). Figure 4C shows comparison of dose volume histograms (DVHs) for a prostate patient (plans shown in Figure 4B). Solid curves represent the plan with shifts, and dashed curves represent the plan with no shift with maximum positional offsets.

**Figure 4A:**
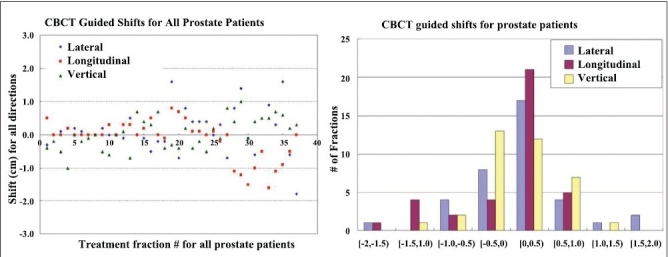
The magnitude of CBCT-guided shifts for all fractions of 8 prostate patients; 68% of shifts are within ±0.5 cm range

**Figure 4B:**
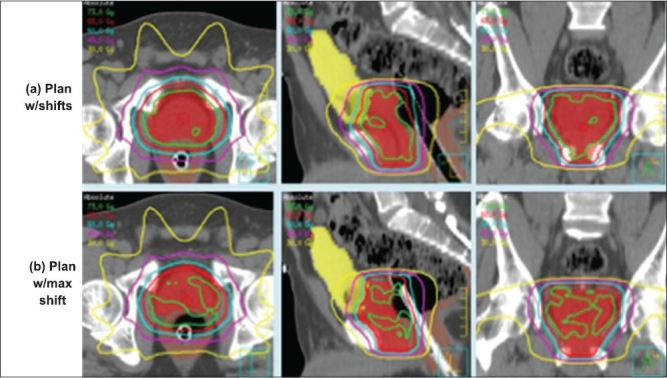
Comparison of isodose distributions for (left to right - transverse, saggital and coronal planes) a prostate patient with IMRT plan in 28 fractions: (a) plan with shifts; (b) plan with maximum offsets (0.0, 0.3, -0.6)

**Figure 4C:**
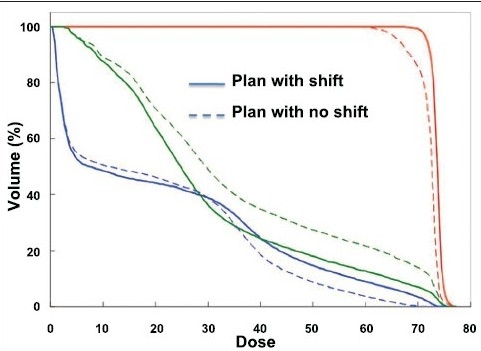
Comparison of DVH for (three regions of interest shown in red green and blue) a prostate patient (plans shown in Figure 4B).

### Impact on tumor control

To further analyze the potential impact on tumor control, tumor control probability (TCP) and target equivalent uniform dose (EUD) were calculated for all of the 8 patients with maximum positional offsets. Our results showed that for cases without daily shifts, the TCP was lower for all 8 patients. For 3 of the patients, the EUD was much lower than the prescribed dose, resulting in a marked reduction in TCP. We believe daily CBCT-guided shifts are necessary for better tumor control. While it is impossible to determine what will lead to failure for a specific patient, it is clear that any setup errors higher than about 0.8 cm can result in a significant reduction in EUD and thus substantially reduce TCP.

**Figure 5:**
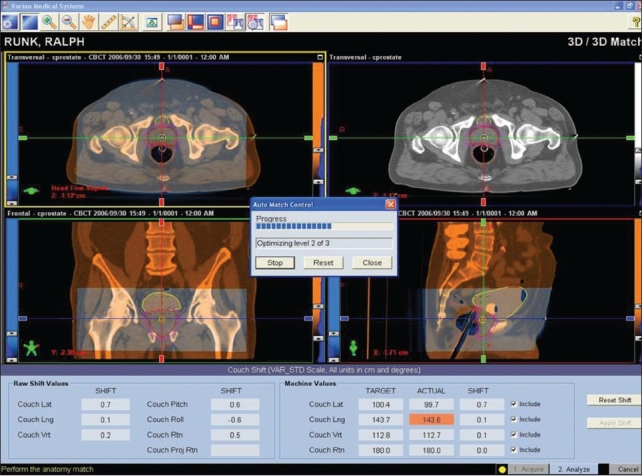
Auto-matching for positioning of a patient based on volumetric CBCT image taken prior to treatment

**Figure 6:**
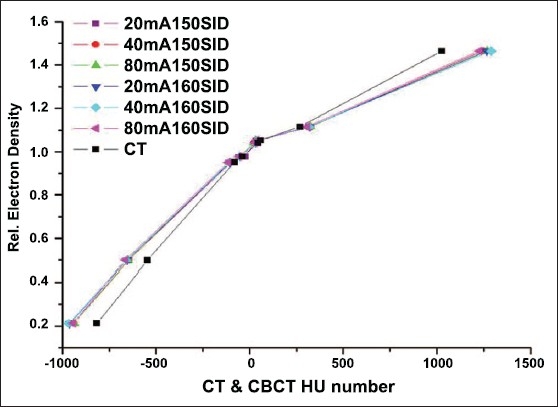
CT density calibration curve for full phan (Head d-phan) mode of CBCT (all possible modes of scanning), along with conventional CT

**Figure 7:**
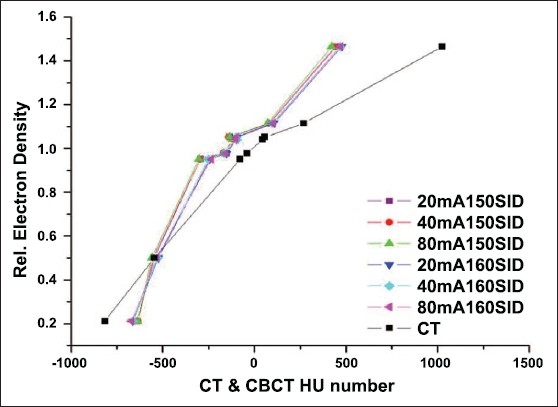
CT density calibration curve for half phan (body d-phan) mode of CBCT (all possible modes of scanning), along with conventional CT

**Figure 8:**
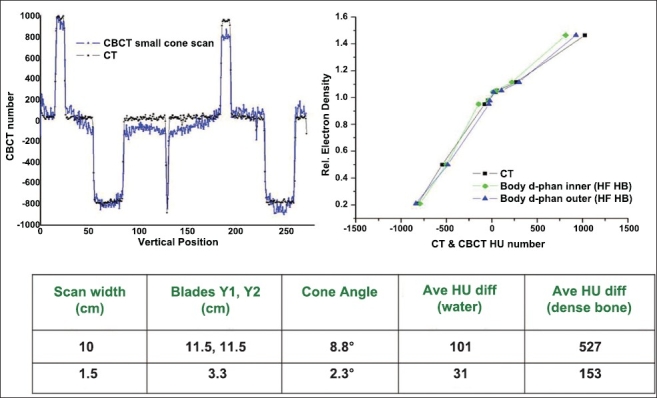
Reducing the cone angle significantly reduces the scatter; thus more uniform HU and better image quality can be achieved

**Figure 9:**
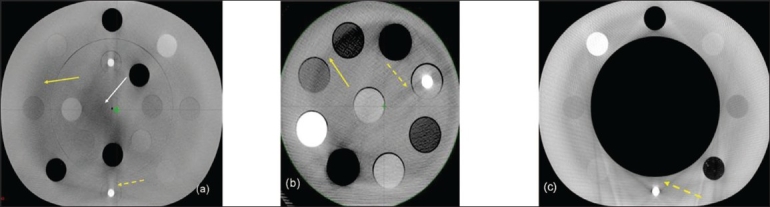
CBCT phantom images showing ring, streaking and cupping artifacts

Online volumetric CBCT matching with planning CT can be expedited using auto-matching algorithms available in some of the commercial systems, as shown in Figure 5. The image sets obtained from these modalities if carefully quantified can lead to dramatic improvement in the quality assurance processes employed in radiation therapy. Both MVCT- and CBCT-based images can be used for dose re-computation for implementing the dose-adaptive radiotherapy. Proper CT density calibration curves are needed prior to the implementation of dose-adaptive radiotherapy. CBCT has 2 modes of scanning, namely, full fan mode (head d-phan) and half fan mode (body d-phan). A separate CT density calibration curve is required for both the scanning modes for all the settings available, as shown in Figures 6 and 7. Moreover, average CT number can vary with cone angle due to scatter, as shown in Figure 8. Although these new technologies demonstrate the very good promise for achieving a precise goal of radiotherapy, there are some inherent problems to be solved. Ring artifacts due to detector inadequacy, streaking artifacts due to beam hardening and scatter and cupping artifacts due to the scattering and beam hardening, as shown in Figure 9. Similarly, MVCT number-to-density calibration is required in order to implement dose-adaptive radiotherapy.

### Resources for patient dose verification

There are several national and international organizations playing pivotal roles in patient dose verification. Bhabha Atomic Research Center (BARC) in India provides the ionization chamber calibration services and also an annual review of regulatory compliance for the radiation oncology facilities in the country. International Atomic Energy Agency (IAEA) conducts the inter-institutional thermo-luminescent dosimeter (TLD) comparison in order to facilitate the comparisons of treatment protocols for better patient care. Similarly there are two such organizations in the United States — Radiologic Physics Center (RPC) and Regional Calibration Laboratories (RCLs) — working towards the same goal. They provide calibration services for ionization chambers, TLD comparisons and phantom designs and benchmarking for advanced treatment-delivery techniques. Advanced Technology Integration Committee (ATIC) and American Association of Physicists in Medicine (AAPM) assist in standardizing the dosimetry protocols through task group reports. Radiation Therapy Oncology Group (RTOG) formulates clinical protocols for newer treatment modalities and delivery techniques.

### Tools for achieving dose verification

**Figure 10:**
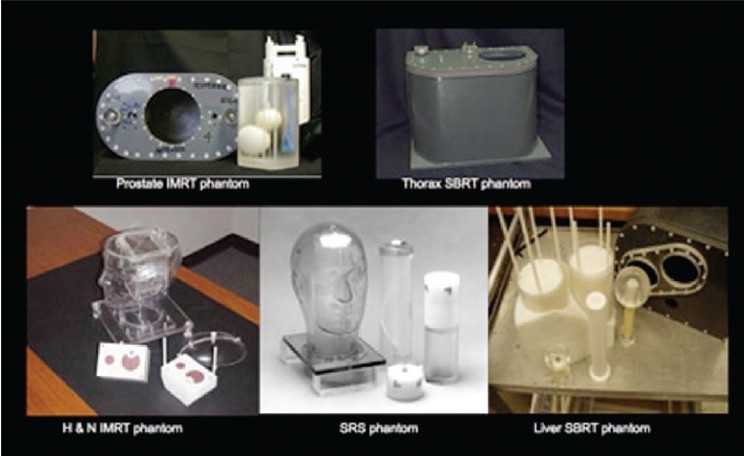
RPC QA phantoms

**Figure 11:**
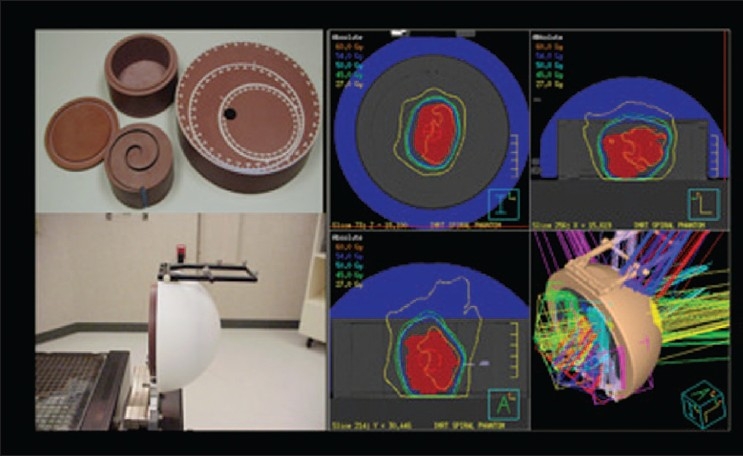
Spiral phantom-based stereo dose verification setup and dose distribution

**Figure 12:**
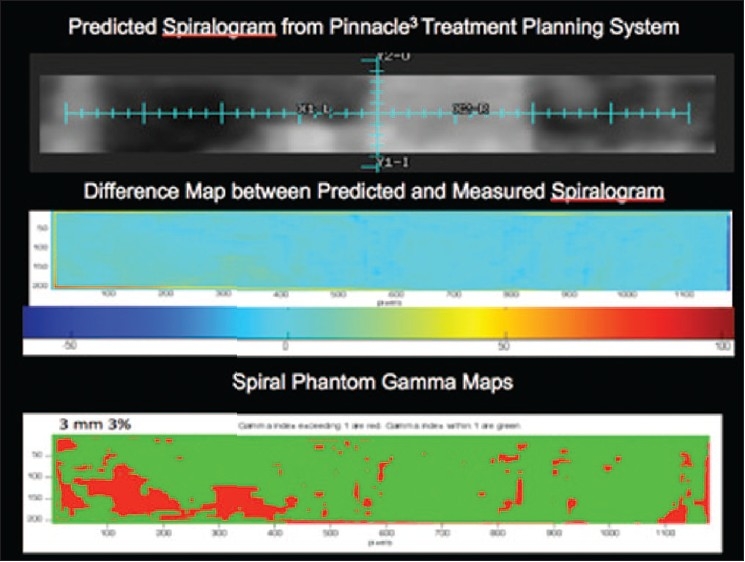
Spiral phantom film analysis: for an IMRT treatment: predicted dose map, dose difference map and gamma map

**Figure 13:**
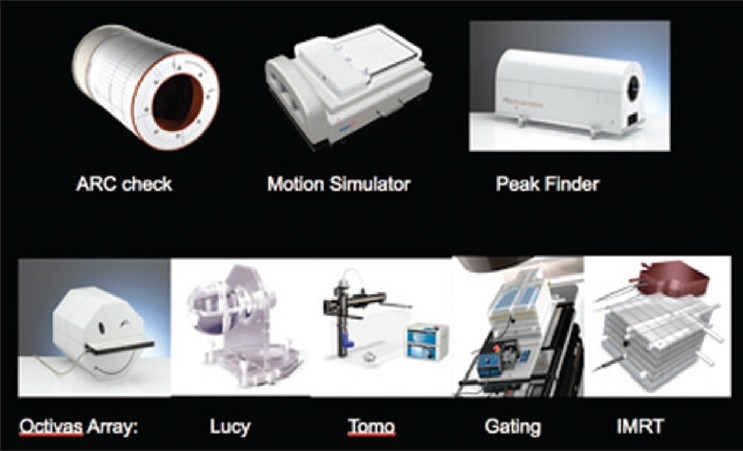
Radiotherapy dosimetry QA phantoms — top row: ArcCHECK, motion simulator, peak finder; bottom row: Octivas array, Lucy, Tomo, gating and IMRT

**Figure 14:**
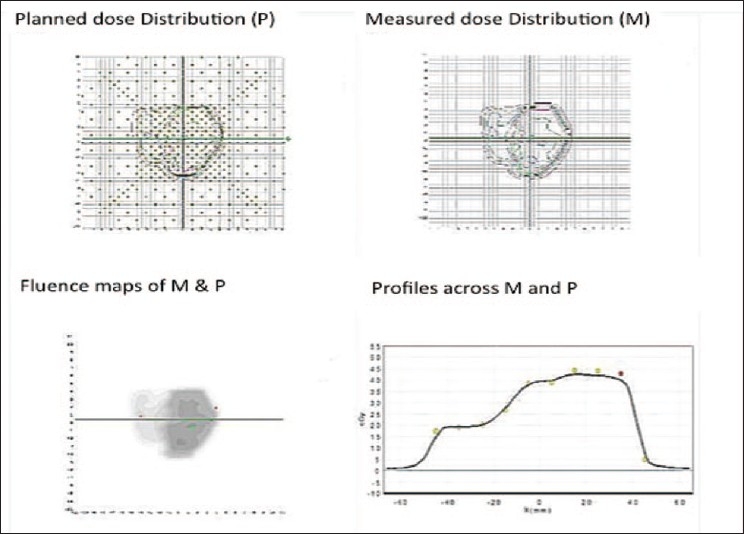
Mapchek-based dose verification

**Figure 15:**
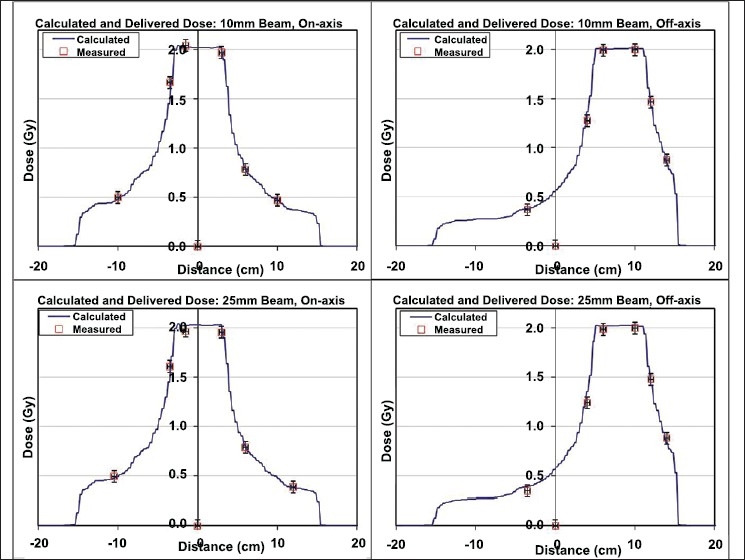
Tomotherapy beam — on and off axis profiles

**Figure 16:**
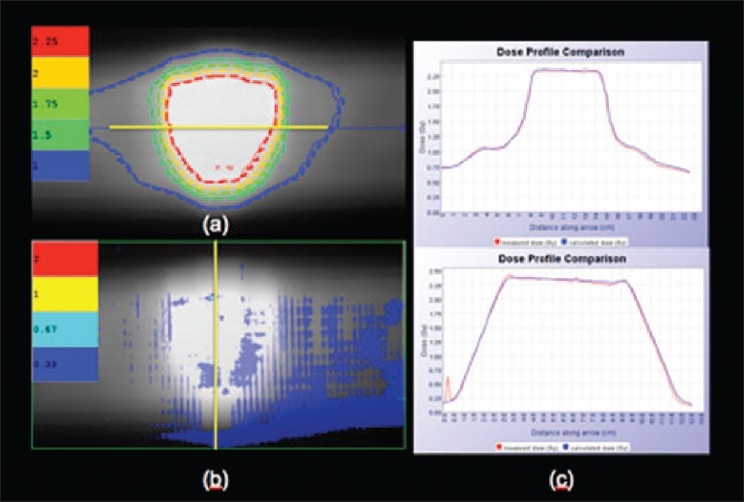
Tomotherapy delivery quality assurance — (a) Tomo-calculated/ measured dose (Gy) contours comparison, (b) Tomo gamma map (3 mm, 3%), (c): Tomo horizontal and vertical profiles

There are several tools for achieving dose verification in radiotherapy. With the advent of more advanced treatment-delivery techniques, some of them are less commonly used due to either their inadequacy for 3D dose verification or uncertainties in analysis procedure. On the other hand, some of the old tools such as TLD and radiochromic film for dose verification still have place in radiotherapy departments. The Radiologic Physics Center (RPC) suggests that the precision of the TLD is ±3%, and the localization precision from the film is 2 mm.[4] TLD and film dosimetry are often used by RPC to monitor institutions participating in the national clinical trials. Examples of the phantoms used by RPC are shown in Figures 10–12, which show a spiral phantom and volumetric sampling of data using film in this spiral phantom.[5] Some additional planar and volumetric data-acquisition devices are shown in Figure 13. Most commonly used tools are electronic portal imaging devices (EPID), tomotherapy detector array, diodes for *in vivo* dosimetry, gel dosimetry system, metal oxide semiconductor field effect transistor (MOSFET) detectors and wireless sensors for remote real-time dose verification.[6,7] Examples of evaluation data from Mapchek, tomotherapy detector arrays and delivery quality assurance are given in Figures 14–16. EPID and all other electronic detectors require careful calibration and evaluation of dose rate, energy and long-term stability. These factors can easily contribute to errors greater than 5%.

### Motion management considerations

Motion management in radiotherapy is a very broad and complex topic. It can significantly modify the planned treatment delivery. An overview of some of the dose verification challenges is provided. Quality assurance for these techniques is challenging and would require the following:

- Evaluation of temporal factors in treatment gating
- Synchrony of tracking device and linear accelerator
- Multiple measurements to determine interplay
- Implementation of algorithm to determine expected motion-corrected dose
- Breathing reproducibility
- Measurement of delivered dose to a moving target

Intrafraction organ motion is mainly due to patient breathing. It primarily compromises the treatment in thorax and abdomen regions. Several techniques, including breath holding, respiratory gating and beam tracking/ synchronized delivery, have been developed or are under development. The implementation of these techniques lacks adequate dose verification tools. Additionally, these techniques demand a robust quality assurance protocol. Some of the related issues are dealt in AAPM's task group #76. So far there is no commercial system available for quality assurance, but some in-house efforts are in place. One of these is designing a sliding platform that can be programmed to move with various periodic and aperiodic trajectories that simulate a patient's respiratory cycle. The amplitudes and frequencies of the respiration can be varied to represent typical patient breathing patterns. To date all the respiratory gating systems are based either on internal tumor motion surrogate or external marker tumor surrogates. Most of the systems in United States and the developing world are based on external respiratory motion surrogates such as markers placed on the surface of the patient's chest or abdomen. The fact that tumor position itself is derived from external breathing signals is the main cause of the error in such treatments. To date there is no universal correlation function giving the consistent tumor position during the treatment. Every treatment contributes to intrafractional and interfractional errors. The objective of breath holding during radiation therapy is to achieve the same target position between fields during a single treatment fraction and between fractions. One commonly used device is a spirometer to measure airflow. Some clinical applications have used the Real-time position management (RPM) system to monitor voluntary breath-hold.[8,9] For spirometry systems, the respiratory signal is time-integrated airflow; thus, the breath-hold is measured in terms of increased lung volume from the baseline position. The RPM system measures abdominal displacement relative to a baseline at end exhalation. A reproducible baseline is an essential first step to achieving reproducible breath-holds. Similar to respiratory gating, a second key issue is the accuracy of externally placed breath-hold monitors in predicting internal positions of the tumor and nearby organs. Patient training is an important component of a clinical QA program that uses breath-hold for treatment.[10] It allows the patient to become familiar with the equipment and procedure, and provides an evaluation of the patient's ability to perform reproducible breath-holds. Some assisted breath-hold, or active breathing control, systems use an additional monitor to provide visual feedback; this helps the patient to achieve a steady breathing pattern and to anticipate the onset of the assisted breath-hold. Patient-related QA programs also require the evaluation and monitoring of external-internal correlation. Kilovoltage fluoroscopy can be used for simulation.

Most of the gated therapy treatments use external gating. To ensure an accurate externally gated treatment, the following steps are recommended:

Acquisition of 4DCT for external gating and verification of target position requires a customized phantom that can move sinusoidally in a plane perpendicular to the CT scan plane.[11] The period and amplitude of motion should be adjustable parameters centering on normal patient respiration (4-s period, 1.5-cm peak-to-peak amplitude). Consideration should be given to phantom registration, target types and location, as well as the analytical tools required for performing motion evaluation.

At the time of acquiring 4DCT, the initial reference position must be accurately established, quantified and used during treatment planning and delivery. Maintaining a constant tumor home position during treatment delivery is also required. The target should always be at the same position when the beam is turned on. During the treatment delivery, tumor positions corresponding to the gating window should be measured and compared with the initial reference position.

We use real-time position management (RPM) respiratory gating systems developed by Varian Medical Systems, Inc. (Palo Alto, CA) and in-house built spirometer-based system for tumor motion management.[12]

**Figure 17:**
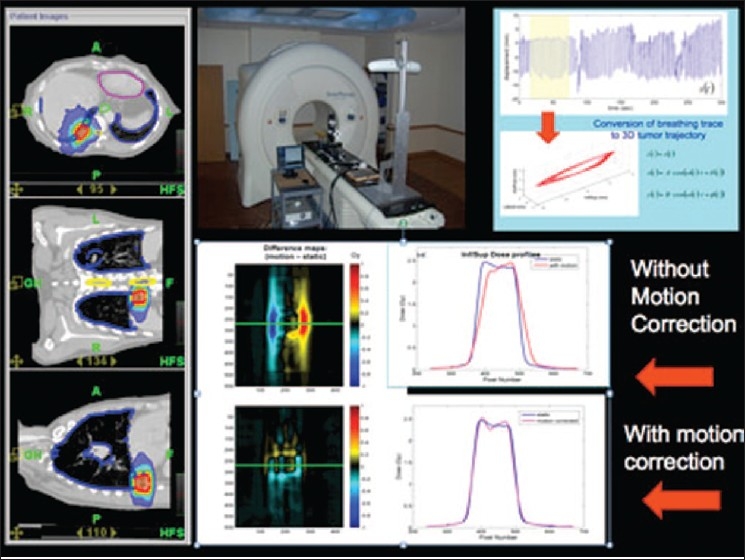
Experimental validation setup for real-time motion-adaptive optimization-guided tomotherapy delivery, breathing trace and tumor trajectory, motion-corrected and motion-uncorrected fluence profiles

We are also considering a noninvasive approach for motion management during tomotherapy. It uses a real-time fiducial external infrared marker for tracking patient breathing and updates a pre-optimized sinogram for motion compensation. A case study is described to illustrate this approach. A lung cancer patient previously treated with tomotherapy was selected for this study. Experimental set up used for treatment delivery and the PTV in all planes is shown in Figure 17. Treatment plan parameters were FW = 1.05 cm, pitch = 0.143, mod factor = 2.0, gantry period = 18; a prescribed dose of 60 Gy (2 Gy × 30 fractions) was delivered. A 4DCT was performed prior to the treatment using spirometer trace for inferior/superior motion. Lateral motion and A/P motion were assumed to be sinusoidal, and 3D target trajectory was generated for use with motion phantom. Motion-corrected and motion-uncorrected dose distributions and profiles are also shown in Figure 17.

For 4DCT, the largest source of error is irregular patient respiration. It causes artifacts in image reconstruction and re-binning. Therefore QA efforts for 4DCT should assure reproducible patient breathing pattern during the scanning process. Achievement of acceptable tumor localization accuracy should be the primary objective of all external gating systems. QA efforts should emphasize the use of external breathing surrogates. Breath-hold treatment techniques are beneficial in some clinical applications, where dose-limiting organs can be moved away from the treatment volume. They are also effective when short-term tumor immobilization is desirable. Frequent imaging throughout treatment is important for monitoring of interfractional variations. Ideally, noninvasive, breathing-synchronized treatment deliveries are preferable. The process of development of these techniques is ongoing.

## Conclusion

New therapeutic strategies are aimed at increasing the efficacy of therapy, while at the same time, ensuring similar or lessened toxicity. There has been manifold development in all aspects of imaging, target localization, patient immobilization, treatment planning and delivery. The complexity of performing these procedures has escalated. The tools for verification are being developed but are yet to provide real-time treatment delivery verification. Even though prior to treatment delivery, verifications in solid phantoms are performed, these do not necessarily guarantee foolproof delivery. The question still remains: have we really delivered the planned treatment? While some of the current processes of quality assurance are valid for many of the devices and software systems in radiation therapy and computer-controlled therapy, these are not necessarily adequate for image-based planning, image-guided therapies. In these areas, additional scientific investigations and innovative approaches are needed to mitigate error and reduce risk to the patients.
